# Post-Transplantation Early Blood Transfusion and Kidney Allograft Outcomes: A Single-Center Observational Study

**DOI:** 10.3389/ti.2022.10279

**Published:** 2022-03-18

**Authors:** Kahina Khedjat, Rémi Lenain, Aghilès Hamroun, Dulciane Baes, Isabelle Top, Myriam Labalette, Benjamin Lopez, Marine Van Triempont, François Provôt, Marie Frimat, Jean-Baptiste Gibier, Marc Hazzan, Mehdi Maanaoui

**Affiliations:** ^1^ Department of Nephrology, CHU Lille, Lille, France; ^2^ INSERM UMR 1246 -SPHERE, Nantes University, Tours University, Nantes, France; ^3^ Clinical Epidemiology Team, CESP, Centre for Research in Epidemiology and Population Health, Inserm, Paris-Saclay University, Versailles Saint Quentin University, Villejuif, France; ^4^ CHU Lille, Institut d’Immunologie, Bd du Professeur Jules Leclercq, Lille, France; ^5^ Lille University, Regional and University Hospital Center of Lille, Lille, France; ^6^ Laboratoire de Biologie Médicale, CH Dunkerque, Dunkerque, France; ^7^ Department of Pathology, Pathology Institute, Inserm UMR-S1172 Lille, JPARC-Jean-Pierre Aubert Research Center, Team “Mucins, Epithelial Differentiation and Carcinogenesis”, Lille, France; ^8^ Univ. Lille, Inserm, CHU Lille, Institut Pasteur Lille, U1190-EGID, Lille, France

**Keywords:** kidney transplantation, allograft failure, graft loss, donor specific antibody, blood transfusion

## Abstract

The association between blood transfusion and the occurrence of *de novo* HLA donor specific antibodies (DSA) after kidney transplantation remains controversial. In this single-center observational study, we examined the association between early blood transfusion, i.e. before 1-month post-transplantation, and the risk of DSA occurrence, using Luminex based-methods. In total, 1,424 patients with a minimum of 1-month follow-up were evaluated between January 2007 and December 2018. During a median time of follow-up of 4.52 years, we observed 258 recipients who had at least one blood transfusion during the first month post-transplantation. At baseline, recipients in the transfused group were significant older, more sensitized against HLA class I and class II antibodies and had a higher 1-month serum creatinine. Cox proportional hazards regression analyses did not show any significant association between blood transfusion and the risk of *de novo* DSA occurrence (1.35 [0.86–2.11], *p* = 0.19), the risk of rejection (HR = 1.33 [0.94–1.89], *p* = 0.11), or the risk of graft loss (HR = 1.04 [0.73–1.50], *p* = 0.82). These data suggest then that blood transfusion may not be limited when required in the early phase of transplantation, and may not impact long-term outcomes.

## Graphical Abstract

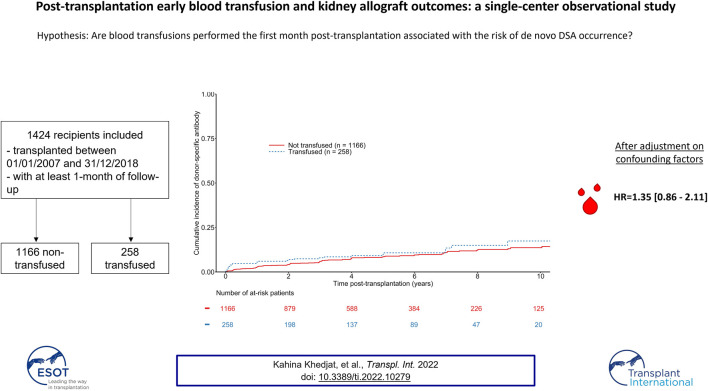

## Introduction

Kidney transplantation (KT) is currently the best treatment option, considering quality of life, life expectancy and cost-effectiveness in end-stage renal disease (1–3). However, there is an increased risk of death during the first months post-transplantation compared to dialysis, owing to surgical and infectious complications (2). This sensitive early post-transplant period brings along increased risks of bleedings due to surgery and anemia of multifactorial origins (infections, inflammation, medications, … ) (4). Early blood transfusion is therefore often necessary, in an era where the age of recipients is constantly increasing, as well as the use of anticoagulant drugs (5).

Blood transfusions are a well-known cause of allogenic sensitization, especially before transplantation. Even though red blood cells are reputed to carry only low levels of Human Leukocyte Antigens (HLA) antigens, blood transfusions also bring few lymphocytes or platelets which may carry class I or class II HLA molecules (6). This antigen exposure to the immune system causes the generation of long-lived alloantibody-producing memory B cells (7) and anti-HLA antibodies. This process is dose-dependent, as the level of pre-transplant sensitization is correlated with the number of pre-transplant transfusions (8). Early blood transfusion is frequent after kidney transplantation, and concerns up to 40–60% among recipients (9–12). Considering its impact, the interrelationship between early blood transfusion allogenic exposure, *de novo* Donor Specific Antibodies (DSA) formation and allograft outcomes is less clear as previous reported cohorts provide contradictory results (9–11). Furthermore, the detection of DSA has greatly evolved over time thanks to Luminex-based methods, and there is a lack of large-scaled studies which examined the link between transfusion and *de novo* DSA occurrence using Luminex.

Our objective was then to examine the impact of post-KT early blood transfusions on *de novo* DSA formation, using Luminex-based methods, in a large cohort of renal transplant recipients. We also evaluated the impact of post-KT blood transfusions on the risk of biopsy-proven acute rejection (BPAR) and graft failure.

## Methods

### Data Source and Ethical Statement

This single-center observational study was performed according to Istanbul Declaration, as well as the Helsinki Declaration ethical guidelines. The study data were collected from *Agence de la Biomédecine*—a state agency that coordinates and administers organ procurement in France—and completed with the recipient medical records. No organs were procured from prisoners. The French legislation stipulates that registry-based research is an integral part of outcome assessment for solid organ transplantation and is exempt from Institutional Review Board approval. All participants provided their informed consent. Patients and laboratory data were pseudonymized and registered according to the French data protection registry (CNIL), referenced #DEC19-054.

### Population

This study included all consecutive adult recipients who had undergone kidney transplantation from January 2007 to December 2018, at the Lille University hospital (Lille, France), with at least 1 month of follow-up. Follow-up terminated in December 2019. Recipients with active or passive desensitization protocol before transplantation were not included as well as patients who previously received transplantation from another organ or a combined transplantation. Patients with lack of information regarding post-transplantation HLA antibody testing were excluded.

### Exposure

Blood transfusions were exhaustively registered thanks to the eTRACELINE software (Mak-System^®^), which identifies the number, the nature and the time of every blood transfusion at the Lille University Hospital. Only ABO-compatible transfusions were performed in the cohort. No information regarding other blood group systems were collected (e.g., rhesus, MNS system, Kell system or others). Only leukocyte-depleted packed red cells were transfused, according to French Laws regarding the risk of infectious agents’ transmission. Early-blood transfusion was then defined as any recipient who benefitted from at least one blood transfusion before 1-month post-transplantation.

### Post-Transplantation Management

The immunosuppressive regimen consisted in an induction therapy (basiliximab or thymoglobulin) and a maintenance triple drug treatment (tacrolimus, mycophenolate mofetil and steroids) for all recipients. Tacrolimus was started at 0.15 mg/kg/d, then adapted to tacrolimus trough level with a target of 10–15 ng/ml up to day-15, and 6–8 ng/ml thereafter. Daily doses of mycophenolate mofetil were 750 mg twice a day. Steroids were withdrawn at day-7 in first-transplant non-sensitized recipient and progressively tapered to 0.1 mg/kg per day in others. Valganciclovir was administered during the first 6 months post transplantation in cytomegalovirus seronegative patients who received a kidney from a cytomegalovirus seropositive donor. A prophylaxis against Pneumocystis jirovecii (trimethoprim-sulfamethoxazole) was prescribed the first 3 months post transplantation.

### Data Collection and Outcomes

The following donors’ parameters were collected: age, sex, blood type, Body Mass-Index (BMI), living donor, cause of death, cold ischemia time, conservation modality (hypothermic perfusion machine (HPM) or static cold storage), donation after brainstem death (DBD) or after circulatory death (DCD). The following recipients’ baseline parameters were collected: age, sex, body mass index (BMI), blood type, cause of end stage kidney disease (ESKD), type of dialysis, time on dialysis, time on the waiting list, previous transplantation, induction therapy, HLA sensitization, number of HLA mismatches, number of blood transfusion, time of blood transfusion, serum creatinine values, time of graft failure defined as the return to dialysis or pre-emptive retransplantation, time of BPAR, time of death.

Anti-HLA antibodies were routinely tested for every recipient at 3 months, 1 year and every year post-KT. Class I and II anti-HLA antibodies were defined by the presence of class I and II anti-HLA antibodies by the LABScreen Mixed Luminex flow bead assay (One Lambda). In case of positivity, specificities were determined according to the LABScreen Single Antigen Luminex flow bead assay (One Lambda). DSA targeting the A, B, Cw, DR, DQ, and DP antigens, were considered as significant if a minimum of mean fluorescence intensity of 1,000 was reached.

In recipients who required blood transfusion, our local protocol involves additional anti-HLA antibodies testings at Day 15, 21, and 28.

The primary outcome of this study was to determine the association between post-KT blood transfusions and the emergence of *de novo* DSA. Secondary outcomes included the association between blood transfusions and one/the risk of BPAR and two/death-censored graft survival. BPAR was determined according to the Banff classification system at the time of kidney biopsy.

### Statistical Analysis

Baseline variables were compared between transfused and non-transfused patients by chi-square (categorical data) or Student’s t-tests (continuous data). The Aalen-Johansen estimator was used to analyze the cumulative incidence of DSA, BPAR and death-censored graft failure accounting for the competing risk of graft loss and death. Hazard ratios (HRs) and 95% confidence intervals associated with transfusion status were estimated using Cox proportional hazards modeling. A multivariate backward selection procedure was implemented, with a univariate threshold *p* < 0.20 for inclusion. Characteristics known to be associated with graft survival were selected *a priori* to be included in the final model even if not significant (cold ischemia time). Log-linearity and the proportional hazards assumption were tested using a graphical method. Sensitivity analyses included the evaluation of the transfusion status on short term outcomes, i.e. the risk of *de novo* DSA occurrence, death-censored graft loss and rejection at 1-year post-transplantation using logistic regression. A multivariate backward selection procedure was implemented, with a univariate threshold of *p* < 0.20 for inclusion. All analyses were carried out in R, version 3.6.3. Statistical significance was determined by a two-tailed *p* value < 0.05.

## Results

### Study Population and Baseline Characteristics

In total, 1,620 recipients underwent kidney transplantation between January 2007 and December 2018. Among these, 1,424 recipients met the criteria of inclusion and had a functional graft at 1-month post-KT (See Flowchart in Supplementary Figure S1). The median time of follow-up was 4.52 years (first−third quartile: 2.41–7.56 years). Overall, 258 recipients (18% of the cohort), benefitted from at least one transfusion before 1-month post-KT, with a median number of two transfusions (first−third quartile: 2–2). Forty recipients benefitted from more than three transfusions. Transfused recipients were significantly older, sensitized in class I and class II HLA antibodies, and had a longer time on the waiting list compared to non-transfused recipients. Donors from transfused recipients were also significantly older, with higher BMI and longer cold ischemia times. Regarding post-transplant characteristics, thymoglobulin induction was more frequent in transfused recipients and baseline median 1-month serum creatinine was significantly higher in transfused recipients (21.00 mg/L [15.00–27.50] vs 17.00 mg/L [13.00–21.85], *p* < 0.001) (Table 1).

**TABLE 1:** Baseline characteristics between transfused and non-transfused recipients.

	Non-transfused (*n* = 1,166)	Transfused (*n* = 258)	*p*-value
Donor			
Age (years), median (IQR)	52.00 (41.00–62.00)	56.00 (46.00–65.00)	0.001
Living donor, *n* (%)	103 (8.83)	3 (1.16)	<0.001
Sexe (female), *n* (%)	506 (43.40)	90 (34.88)	0.015
BMI (kg/m^2^), median (IQR)	25.46 (22.58–28.54)	26.10 (23.53–29.41)	0.007
Blood type, *n* (%)			0.659
A	473 (40.57)	111 (43.02)	
AB	36 (3.09)	9 (3.49)	
B	107 (9.18)	18 (6.98)	
O	550 (47.17)	120 (46.51)	
Recipient			
Age (years), median (IQR)	51.89 (39.19–60.47)	56.25 (45.26–62.80)	<0.001
First kidney transplantation, *n* (%)	986 (84.56)	204 (79.07)	0.039
Sexe (female), *n* (%)	389 (33.36)	132 (51.16)	<0.001
BMI (kg/m^2^), median (IQR)	24.52 (21.63–27.54)	24.78 (21.75–28.70)	0.137
Blood type, *n* (%)			0.557
A	493 (42.28)	117 (45.35)	
AB	49 (4.20)	12 (4.65)	
B	121 (10.38)	20 (7.75)	
O	503 (43.14)	109 (42.25)	
Type of dialysis			0.380
Hemodialysis, *n* (%)	909 (77.96)	208 (80.62)	
Peritoneal dialysis, *n* (%)	133 (11.41)	30 (11.63)	
Preemptive transplantation, *n* (%)	124 (10.63)	20 (7.75)	
Cause of ESKD			0.818
Glomerulonephritis, *n* (%)	153 (13.12)	40 (15.50)	
Vascular nephropathy, *n* (%)	333 (28.56)	72 (27.91)	
Undetermined, *n* (%)	93 (7.98)	23 (8.91)	
Diabetes, *n* (%)	148 (12.69)	32 (12.40)	
ADPKD, *n* (%)	73 (6.26)	20 (7.75)	
Tubulo-interstitial nephritis, *n* (%)	230 (19.73)	44 (17.05)	
Others, *n* (%)	136 (11.66)	27 (10.47)	
Waiting time on dialysis, median (IQR)	2.11 (1.12–3.71)	2.58 (1.44–4.25)	0.003
HLA sensitization class I, *n* (%)	187 (16.04)	61 (23.64)	0.008
HLA sensitization class II, *n* (%)	203 (17.41)	71 (27.52)	0.001
Transplantation			
Cold ischemia time (h), median (IQR)	15.83 (11.68–20.67)	18.27 (14.08–23.42)	<0.001
Hypothermic perfusion machine, *n* (%)	243 (20.84)	59 (22.87)	0.321
ABDR mismatches, median (IQR)	4.00 (3.00–5.00)	4.00 (3.00–4.00)	0.665
Induction therapy (Thymoglobulin), *n* (%)	695 (59.61)	158 (61.24)	0.008
1-month baseline serum creatinine (mg/L), median (IQR)	17.00 (13.00–21.85)	21.00 (15.00–27.50)	<0.001
Number of transfusions			
1 or 2	—	218 (84.50)	
Over 3	—	40 (15.50)	

ADPKD, autosomal dominant polycystic kidney disease; BMI, body mass index; ESKD, end-stage kidney disease HLA, human leukocyte antigen; IQR, InterQuartile Range.

### Association Between Post-KT Blood Transfusions and Emergence of *De Novo* DSA

**FIGURE 1:**
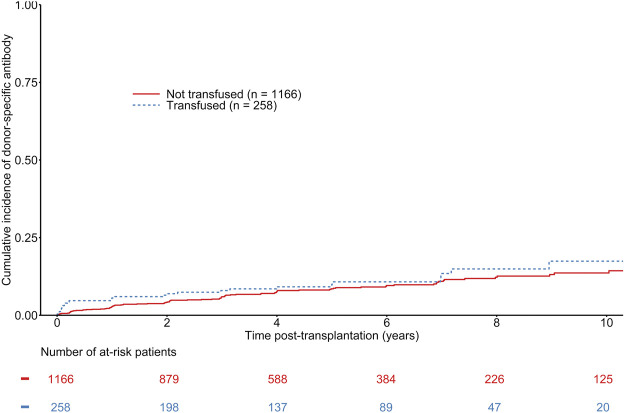
Cumulative incidence of *de novo* donor specific antibodies according to the transfusion status of kidney transplant recipients. Gray-test: *p* = 0.32.

The median time to *de novo* DSA occurrence was 731 days (first−third quartile: 173–1,461 days). The mean number of HLA measurements during follow-up was 5.71 (±3.14) in transfused recipients and 5.3 (±3.36) in non-transfused recipients. A total of 124 patients developed *de novo* DSA, including 28 in transfused recipients. The overall estimated probability of DSA occurrence was 3.22% (CI 95% 2.41–4.29), 6.04% (CI 95% 4.87–7.49), 8.20% (CI 95% 6.75–9.93) at 1, 3, and 5 years respectively. The estimated probabilities of DSA occurrence at 1, 3, and 5 years were 2.73% [CI 95% 1.93–3.86], 5.74% [CI 95% 4.48–7.34], and 8.03% [CI 95% 6.44–9.98] in non-transfused recipient, versus 5.43% [CI 95% 3.25–8.99], 7.49% [CI 95% 4.84–11.50], and 9.09% [CI 95% 6.05–13.54] in transfused recipients (See Figure 1). Multivariable Cox regression models did not show any association between transfusion and *de novo* DSA occurrence (HR = 1.35 [0.86–2.11], *p* = 0.19) (See Table 2). Being highly transfused (i.e. over three transfusions) was also not associated with an increased risk of *de novo* DSA occurrence (HR = 0.93 [0.29–2.97], *p* = 0.90). Furthermore, we did not find any significant difference regarding the nature of DSA (Class I or Class II DSA) according to the transfusion status (Supplementary Table S1). Other independent predictors of *de novo* DSA occurrence included recipient and donor age, HLA class II sensitization, and the number of HLA ABDR mismatches (See Table 2). As a sensitivity analysis, we analyzed the short-term effects of early blood transfusion on *de novo* DSA occurrence at 1-year post-transplantation. Forty-five recipients presented with *de novo* DSA at 1-year post-transplantation. Multivariate logistic regression did not show any association between transfusion and *de novo* DSA occurrence at 1-year (OR = 1.58 [0.79–3.18], *p* = 0.20) (See Supplementary Table S2).

**TABLE 2:** Multivariate Cox regression model for the risk of development of *de novo* DSA.

	*de novo* DSA	
	Multivariate HR [95% CI]	*p*-value
Blood transfusion post-KT (yes vs. no)	1.35 [0.86–2.11]	0.19
1 or 2 blood transfusions	1.43 [0.89–2.30]	0.13
Over 3 blood transfusions	0.93 [0.29–2.97]	0.90
Recipient age (per year)	0.95 [0.93–0.97]	< 0.01
Donor age (per year)	1.03 [1.01–1.05]	< 0.01
HLA sensitization class II (yes vs. no)	1.81 [1.18–2.80]	0.01
ABDR mismatches (>4 vs. ≤ 4)	1.33 [1.14–1.55]	< 0.01

HLA, human leukocyte antigen; KT, kidney transplantation.

### Association Between Post-KT Blood Transfusions and Biopsy-Proven Acute Rejection

**FIGURE 2:**
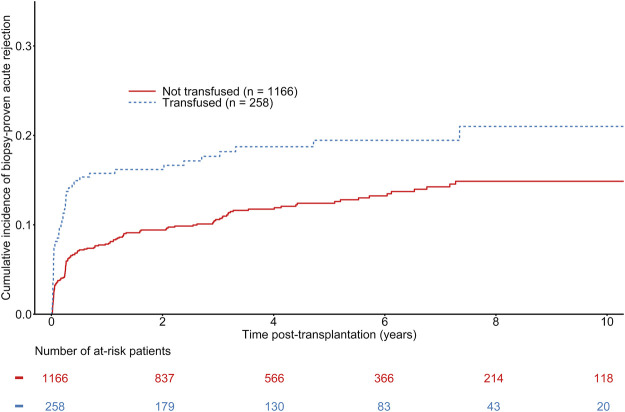
Cumulative incidence of biopsy-proven rejection according to the transfusion status of kidney transplant recipients. Gray-test: *p* = 0.004.

The median time to BPAR onset was 94 days (first−third quartile: 17–475 days). A total of 189 patients were diagnosed with BPAR, including 49 in the group of transfused recipients. The overall estimated probability of BPAR was 7.80% [CI 95% 6.39–9.50], 10.42% [CI 95% 8.76–12.38] at 1 and 3 years respectively. The estimated probabilities of BPAR at 1 and 3 years were 7.80% [CI 95% 6.39–9.50] and 10.42% [CI 95% 8.76–12.38] vs. 15.52% [CI 95% 11.63–20.55] in non-transfused recipient and 17.21% [CI 95% 13.10–22.43] in transfused recipients (See Figure 2). Even though univariate analyses suggested a significant difference between transfused and non-transfused recipients (See Figure 2), adjusted multivariable Cox regression models did not show any association between transfusion and BPAR (HR = 1.33 [0.94–1.89], *p* = 0.11) (See Table 3). Other independent predictors of BPAR involved donor sex, HLA class II sensitization, and 1-month serum creatinine (See Table 3). As a sensitivity analysis, we analyzed the short-term effects of early blood transfusion at 1-year post-transplantation on the risk of BPAR. One hundred and thirty recipients presented with BPAR at 1-year post-transplantation. On the contrary to the long-term analysis, multivariate logistic regression showed an association between transfusion and BPAR at 1-year (OR = 1.63 [1.05–2.52], *p* = 0.03). Other independent variables associated with the risk of BPAR at 1-year remained the same than presented in the Cox model (See Supplementary Table S3).

**TABLE 3:** Multivariate Cox regression model for the risk of biopsy-proven acute rejection.

	BPAR	
	Multivariate HR [95% CI]	*p*-value
Post-KT blood transfusion (yes vs. no)	1.33 [0.94–1.89]	0.11
Male donor	0.75 [0.56–1.00]	0.05
HLA sensitization class II	1.83 [1.32–2.52]	<0.01
1-month serum creatinine (per 0.1 mg/dl)	1.02 [1.01–1.03]	<0.01

BPAR, biopsy-proven acute rejection; HLA, human leukocyte antigen; KT, kidney transplantation.

### Association Between Post-KT Blood Transfusions and Graft Loss

**FIGURE 3:**
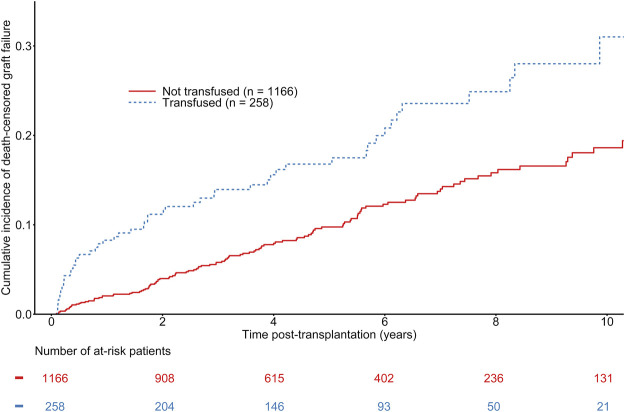
Cumulative incidence of kidney graft failure according to the transfusion status of kidney transplant recipients. Gray-test: *p* < 0.001.

The median time to graft failure was 973 days (first−third quartile: 336–1925 days). A total of 170 patients experienced graft failure during follow-up, including 52 in the group of transfused recipients. The overall estimated probability of death-censored graft failure at 1, 3, and 5 years was 3.15% [CI 95% 2.36–4.22], 7.17% [CI 95% 5.87–8.73], and 10.70% [CI 95% 9.02–12.67], respectively. The estimated probabilities of death-censored graft failure at 1, 3, and 5 years were 2.03% [CI 95% 1.35–3.04], 5.68% [CI 95% 4.42–7.30], 9.44% [CI 95% 7.66–11.61] in non-transfused recipient versus 8.16% [CI 95% 5.40–12.23], 13.61% [CI 95% 9.91–18.54], and 16.25% [CI 95% 12.10–21.63] in transfused recipients (See Figure 3). Even though univariate analyses suggested a significant difference between transfused and non-transfused recipients (See Figure 3), adjusted multivariable Cox regression models did not show any association between transfusion and graft loss (HR = 1.04 [0.73–1.50], *p* = 0.82) (See Table 4). Other independent predictors of graft failure included recipient and donor age, waiting time on dialysis, and 1-month serum creatinine. As a sensitivity analysis, we analyzed the short-term effects of early blood transfusion at 1-year post-transplantation. Forty-four recipients presented with graft failure. Multivariate logistic regression showed a trend of association between transfusion and BPAR at 1-year (OR = 2.02 [0.93–4.40], *p* = 0.08). The other independent variables significantly associated with the risk of death-censored graft failure at 1-year remained the same than presented in the Cox model, except for the waiting time on dialysis (See Supplementary Table S4).

**TABLE 4:** Multivariate Cox regression model for death-censored graft loss.

	Graft loss	
	Multivariate HR [95% CI]	*p*-value
Post-KT blood transfusion (yes vs. no)	1.04 [0.73–1.50]	0.82
Recipient age (per year)	0.98 [0.97–1.00]	0.03
Donor age (per year)	1.03 [1.02–1.05]	<0.01
Waiting time on dialysis (per year)	1.07 [1.04–1.11]	<0.01
Cold ischemia time (per hour)	1.02 [1.00–1.04]	0.09
1-month serum creatinine (per 0.1 mg/dl)	1.06 [1.05–1.06]	<0.01

KT, kidney transplantation.

## Discussion

In this single-center study comprising a large number of KT and a median time of follow-up of 4.52 years, we did not show any association between post-KT early blood transfusions and the occurrence of *de novo* DSA, BPAR or graft failure.

Allorecognition leads to the generation of alloantibodies targeting non-self antigens, i.e. *de novo* DSA (7), which are associated with an increased risk of antibody-mediated rejection and allograft failure (13). As far as blood transfusions are concerned, the risk of induced-alloimmunization seems to have decreased over the last decades (14). A blood product is composed of three distinct parts (1): the desired product, such as red blood cells (RBCs) or platelets (2); excipients (e.g. anticoagulant or residual plasma) (3); residual leukocytes that carry HLA antigens, and in rare cases unexpected residual cells, such as platelets in red blood cells products (15). Leukocytes carry most of the antigenic load in a blood unit, yet the systematic use of leukoreduction process implemented in the late 90’s to fight against Creutzfeldt-Jakob disease transmission dramatically reduced the amount of WBC into blood units (16, 17). As a consequence, the rate of post-transfusion HLA sensitization decreased from nearly 30% of transfused patients to 10–20% depending on studies (18–21). However, even with leukoreduction, the risk of sensitization still persists as erythrocytes constitutively express HLA class I molecules at low levels (22). The risk of transfusion-related sensitization also depends on the immunological history of the recipient. Indeed, transfused kidney transplant candidates with a history of pregnancy or previous transplantation have a higher risk of sensitization after transfusion compared to kidney transplant candidates with a sole history of blood transfusion (23). Moreover, there seems to be a dose-effect, as the level of immunogenicity correlates with the number of administered units (6, 24).

Even though blood transfusion seems to be clearly associated with the risk of HLA sensitization, its impact on allograft outcomes remains unclear. Paradoxically, throughout the early beginnings of solid organ transplantation and before the implementation of cyclosporine, pre-transplant blood transfusion was supposed to be associated with immunomodulatory properties and benefits on renal allograft outcomes (25, 26). Donor-specific transfusion in KT has long been part of routine practices for its supposed ability to prevent post-transplant rejection (27, 28). Animal models also provided evidence of transfusion-related immunomodulation properties owing to the generation of alloreactive CD25^+^CD4^+^ regulatory T cells that prevent graft rejection (29), from both related and unrelated donor blood. Nowadays, even if donor-specific transfusion is no longer used, the potential immunomodulatory properties of blood transfusion question the impact of early blood transfusion after KT.

In our study, we did not find any association between early blood transfusion post-KT and the further risk of *de novo* DSA development, on a large-scaled cohort using Luminex-based methods to identify DSA. On the contrary, HLA mismatches and HLA sensitization were significantly associated with *de novo* DSA and are a well-known risk factors of post-transplantation allosensitization (30, 31). Aging was also associated with the risk of *de novo* DSA occurrence. On the one hand, aging in recipients was associated with a lower risk of allosensitization, which may reflect the aging-related immunosenescence in recipients. On the other hand, aging in donors was associated with an increased risk of *de novo* DSA occurrence, which exhibits the aging-related immunogenicity of kidney donors (32). The transfusion status was also not associated with secondary outcomes such as the long-term risks of rejection or graft failure. Considering the literature, Scornik et al. (9) reported 746 patients transplanted followed for 6 years, including 45% transfused-recipients with 79% of blood transfusions performed during the first month post-KT. There was no significant difference regarding the incidence of rejection episodes or graft loss according to the transfusion status. There was also no difference regarding the frequency of *de novo* DSA between transfused and non-transfused recipients (17% vs. 15%, *p* = 0.67). Verghese et al. (12) reported then a pediatric study of 482, including 44% transfused patients. Among these, 134 recipients could be tested for HLA antibodies using solid-phase based methods, including 82 transfused recipients. In their study, blood transfusion was also not associated with the risks of *de novo* DSA after KT (HR 0.9; 95% CI 0.6–1.4; *p* = 0.65), rejection or graft failure. In the same way, Daloul et al. recently reported their experience of 273 recipients, including 127 transfused recipients before 1-month post-KT. They did not find any difference at 1-year post-KT regarding the incidence of *de novo* DSA using solid-phase based methods (12.8% in transfused recipients and 10.9% in non-transfused recipients, *p* = 0.48) (33), as well as with the risk of rejection or graft loss.

Conversely, Ferrandiz et al. showed opposite results regarding the association between blood transfusion and *de novo* DSA, with one of the largest cohorts studying HLA antibodies using Luminex. Three hundred and ninety non-sensitized kidney transplant recipients were included, of which 250 were transfused during the first year post-KT. 94.8% of them were transfused during the first month post-KT. During the first-year post-transplantation, 18 recipients (7.2%) in the transfusion group developed *de novo* DSA, compared to only one (0.7%) in the nontransfusion group (*p* < 0.0001). This higher prevalence of *de novo* DSA was also associated with a higher incidence of ABMR (15 transfused-recipients (6%) vs two non-transfused recipients (1.4%), *p* = 0.04). However, baseline characteristics significantly differed regarding notably the immunosuppressive regimen, with a higher proportion of transfused recipients treated with cyclosporine. Furthermore, they examined early outcomes as logistic regression at 1-year post-KT revealed that both the use of cyclosporin and blood transfusions were associated with the risk of DSA formation. In our study, both the evaluation of early and long-term outcomes did not find any association with the transfusion status. Yet, we acknowledge that it may be difficult to compare these two monocentric studies, as far as local practices, e.g. regarding the immunosuppressive regimen management, may influence the results. Recently, Hassan et al. reported a cohort of 1,104 recipients including 667 transfused recipients. 88.9% of blood transfusions were performed before 1-month post-KT. Blood transfusion was significantly associated with the development of *de novo* DSA (transfusion received: HR = 1.49 [1.10–2.04], *p* = 0.01) and graft failure (transfusion received: HR = 1.85 [1.19–2.77], *p* = 0.005). However, the prevalence of blood transfusion was surprisingly high, which could be linked to the baseline characteristics of the overall cohort (not provided). Nevertheless, they provided novel data dealing with the analysis of shared transfusion and kidney donors’ alloantibodies in transfused recipients. They analyzed a subgroup of 86 transplant recipients who received transfusion from 244 blood donors. Overall, 61.5% of transfused recipients developed *de novo* transfusion specific antibodies (TSA), of which 46.7% shared HLA antibody specificity with a DSA response in the recipient (DSA+/TSA+). DSA+/TSA + recipients had an increased risk of allograft loss or rejection compared to recipients with only TSA or DSA. This may suggest a need of blood donor HLA matching in kidney transplant recipients.

Compared to the existing literature, our study has two main strengths. First, we provide one of the largest cohort of recipients screened for HLA antibodies during their whole follow-up, combined with their transfusion status. Second, this cohort benefitted from a long-term follow-up with the evaluation of reliable time-dependent outcomes. Still, our findings need to be interpreted in the context of some caveats. Indeed, the retrospective nature of the study could be associated with information bias. Then, it is also limited by the lack of information regarding the hemoglobin levels. Post-KT anemia is indeed known to be associated with mortality and graft loss (34, 35). Yet, our primary outcome is focused on the emergence of *de novo* DSA and no association between anemia and *de novo* DSA has been reported to date. Thus, it does not seem to constitute a confounding factor. Finally, there are significant baseline differences between transfused and non-transfused recipients that should be considered to interpret our results. Donors from transfused recipients were significantly older, with longer cold ischemia times. Transfused recipients were significantly older, sensitized against HLA antibodies, more frequently treated with thymoglobulin induction and had a significant worse graft function at 1-month post-transplantation. These baseline differences may explain why univariate and short-term analyses revealed differences concerning rejection and graft failure. However, after adjusting on confounding factors on long-term analyses, transfusion was no longer associated with any of those outcomes. To be noted, as far as our primary criteria of judgement is concerned, none of our analyses, i.e. short-term or long-term, univariate and multivariate, revealed an association between transfusion and *de novo* DSA occurrence.

Ultimately, even if on a global scale we did not find any association between transfusion and the development of *de novo* DSA, it does not mean that this correlation does not exist at an individual level, as suggested by Hassan et al. The risk of allosensitization should be kept in mind, and strategies of HLA matching between blood and kidney donors may be of interest in the next few years. However, our data provide evidence that transfusion should not be limited in the early period post-KT when required.

## Conclusion

In our large-scaled cohort of kidney transplant recipients, we did not find any association between post-KT early blood transfusions and the development of *de novo* DSA, nor the risks of rejection and graft failure.

## Data Availability

The raw data supporting the conclusion of this article will be made available by the authors, without undue reservation.

## Abbreviations

ABMR, Antibody-Mediated Rejection; BMI, Body-Mass Index; BPAR, Biopsy-Proven Acute Rejection; DBD, Donation after Brainstem Death; DCD, Donor after Circulatory Death; DSA, Donor Specific Antibodies; ESKD, End-Stage Kidney Disease; HLA, Human Leukocyte Antigen; HPM, Hypothermic Perfusion Machine; HR, Hazard-Ratio; IQR, Interquartile Range; KT, Kidney Transplantation; RBC, Red Blood Cell; TSA, Transfusion Specific Antibodies; WBC, White Blood Cells.
