# Supplementary material for: Post-Transplantation Early Blood Transfusion and Kidney Allograft Outcomes: A Single-Center Observational Study
Source: Transpl Int. 2022 Mar 18;35:10279. doi: 10.3389/ti.2022.10279 (PMC8971186; doi:10.3389/ti.2022.10279)
Supplement: Supplementary file 1 [file DataSheet1.PDF]

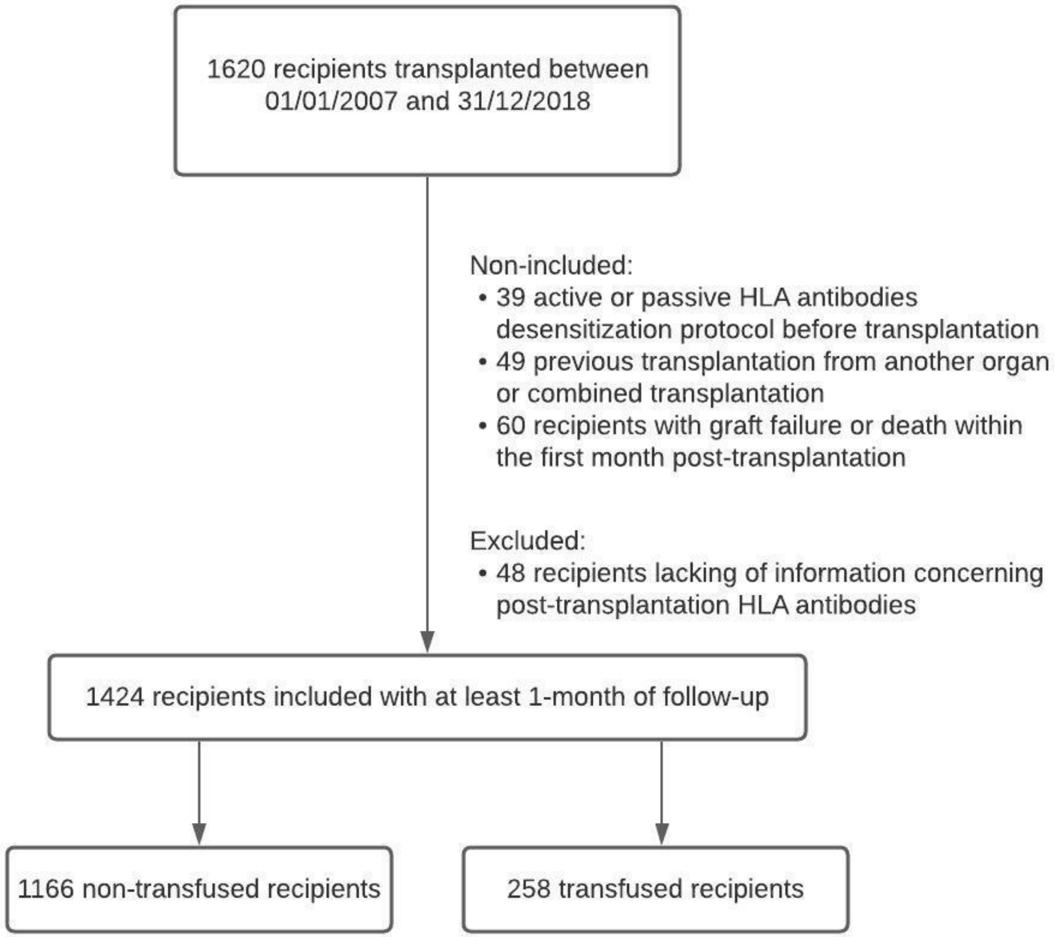

Supplementary Figure 1. Study flowchart.

Supplemental Table 1. Characteristics of Donor Specific Antibodies according to the transfusion status.

	Non-transfused (n=96)	transfused (n=28)	*p*-value
HLA Class I	49 (51.0)	18 (64.3)	0.307
A	27 (28.1)	10 (35.7)	0.591
B	24 (25.0)	11 (39.3)	0.215
Cw	14 (14.6)	2 (7.14)	0.521
HLA Class II	59 (61.5)	17 (60.7)	1.000
DP	10 (10.4)	2 (7.14)	1.000
DQ	40 (41.7)	10 (35.7)	0.729
DR	17 (17.7)	5 (17.9)	1.000

Supplemental Table 2. Logistic regression model for the risk of development of *de novo* DSA at 1-year post-transplantation.

	de novo DSA	
	Multivariate OR [95%CI]	*p*-value
Blood transfusion post-KT (yes vs no)	1.58 [0.79 - 3.18]	0.20
HLA sensitization class II (yes vs no)	1.81 [1.18 - 2.80]	< 0.01
Hypothermic perfusion machine (yes vs no)	2.70 [1.42 - 5.10]	< 0.01

HLA= Human Leukocyte Antigen; KT = Kidney Transplantation.

Supplemental Table 3. Logistic regression model for the risk of development of biopsy-proven acute rejection at 1-year post-transplantation.

	BPAR	
	Multivariate HR [95%CI]	*p*-value
Blood transfusion post-KT(yes vs no)	1.63 [1.05 - 2.52]	0.03
Male donor	0.67 [0.46 - 0.98]	0.04
HLA sensitization class II (yes vs no)	2.28 [1.52 - 3.44]	< 0.01
1-month serum creatinine (per 0.1 mg/dL)	1.02 [1.01 - 1.04]	< 0.01

BPAR= Biopsy-Proven Acute Rejection; HLA= Human Leukocyte Antigen; KT = Kidney Transplantation.

Supplemental Table 4. Logistic regression model for the risk of death-censored graft failure at 1-year post-transplantation.

	Graft loss	
	Multivariate HR [95%CI]	*p*-value
Blood transfusion post-KT (yes vs no)	2.02 [0.93 - 4.40]	0.08
Recipient age (per year)	0.96 [0.93 - 0.99]	0.02
Donor age (per year)	1.06 [1.03 - 1.10]	< 0.01
Cold ischemia time (per hour)	0.96 [0.90 - 1.01]	0.14
1-month serum creatinine (per 0.1 mg/dL)	1.08 [1.06 - 1.10]	< 0.01

KT = Kidney Transplantation.
